# Items of General Information

**Published:** 1895-03

**Authors:** 


### Medical News and Miscellany.

**Dr. J. Q. Burton** has removed from Jacksonville, Texas, to Mt. Selmon, Texas.

**Dr. B. W. Palmer,** for some years editor of the Detroit *Medical Age*, died at Nyack-on-the-Hudson, January 4.

**In a recent fire** in Jewett, Texas, Dr. W. T. Evans lost his office, books, instruments and stock of drugs. No insurance.

**Dr. Emory Lanphear** has resigned from the faculty of the St. Louis College of Physicians and Surgeons, and also from the editorship of the college organ, the *St. Louis Clinique.*

**The Texas Medical College** (Medical Department, University of Texas).—The legislature has made an appropriation of $40,500 for the medical branch, in addition to the $35,000 for the University proper.

**Sic Transit.**—The Wooten Bill to Regulate the Practice of Medicine came up in the Texas Senate on March 3d as the special order of the day, and was, on motion of Senator Steele, indefinitely postponed.

**The French Government** has made a decree shutting out American cattle on the alleged ground of pleuro-pneumonia. Minister Eustis has entered a protest. This will throw it into the courts, and act as a stay, at least for some months.

**Fort Worth Medical College.**—A letter from Prof. E. J. Beall, M. D., of the Medical Department Fort Worth University, reports "about seventy good students" in attendance on lectures, and good grounds for expecting 150 next session. Good!

**Dr. G. W. Cain,** of Elgin, an old resident physician of that place, a member of the State Medical Association and of the Austin District Medical Society, died at his home on the 7th of February, ult., of "heart failure," says the *Texas Sanitarian*.

---

**Singular Fact.**—The total amount necessary to run the great State of Texas is $2,750,000, that being the amount, in round numbers, of the estimate on which the appropriation will be based. Texas has, in round numbers, 2,750,000 inhabitants, and produces 2,750,000 bales cotton.

---

**Died,** February 21st ult., Dr. Grace Danforth, at her home in Granger, Texas. Dr. Danforth was a talented lady physician, who, had she lived, would have achieved distinction. She was sister of Dr. C. A. Danforth, whose early demise the JOURNAL was called upon to chronicle a few months ago.

---

**The quarantine steamer,** "Bessie Ross," purchased by Dr. Rutherford when he was State Health Officer, and named by him, and which has for four years been lying unused at the Harrisburg wharf, has been sold by State Health Officer Swearingen, for $2500, by special act of the legislature authorizing sale. She cost $15,000.

---

**A Fellowship in Pharmacy,** Chemistry and Pharmacology, to be known as the "Stearns Fellowship" has been established at the University of Michigan at Ann Arbor, and endowed by Frederick Stearns & Co. by $600 donation. Messrs. Stearns & Co. only recently presented the University with an art collection comprising hundreds of paintings of Japanese fishes.

---

**We ought to have** an inspector of all drugs and medicines of tha patent kind, in addition to a strict law regulating their sale. In Boston, recently, there was brought to the notice of the board of health a patent "face-bleach," consisting of a four ounce mixture, containing 27 grains of corrosive sublimate. We don't know if it would bleach, but it would surely blister.

---

**The Senate** Committee have recommended $42,000, the amount estimated and asked for by the State Health Officer as the appropriation for the quarantine department. The joint committee has not yet reported. Should this appropriation carry, $2000 of it will be devoted by the State Health Officer to bacteriological research. The House Committee has reported $40,000.

---

**The Texas State Medical Association**—let it be remembered—meets in Dallas on the 4th Tuesday next month—April. If the Secretary should send in the program and announcement in time, they will appear in our April number. Meantime the papers for the Section of Practice should be sent to Dr. J. W. Carhart, LaGrange, Chairman, or to Dr. J. H. Frey, Dallas, Secretary.

---

**Castration for Hypertrophy of the Prostate.**—In our February number we published Dr. J. William White's paper on this subject. Dr. L. M. Green, in *Lancet-Clinic*, reports another case successfully operated upon, in addition to those reported in Dr. White's paper. The success of the operation is considered established and it will doubtless be called "the White operation."

---

**A funny question** has been sprung: Is there any sound where there are no ears? Does the falling of a tree, for instance, make a noise when no one hears it? We invite a discussion through our columns; it will give readers something to puzzle over as they jog along over curduroy roads "dollar-a-mile" (on paper), and relieve the strain of figuring how one is going to make the *promises* of his patrons, pay his grocery bill.

---

**The Medical Examiner** is a monthly journal devoted to the exposition of the theory and practice of physical diagnosis, and to the study of disease. It is published in the interest of medical examiners for the Army and Navy, for Life Insurance, for Accident Insurance, for Masonic Insurance, for Police Departments, for Fire Departments, for the Pension Bureau, for the Civil Service and for Railroads, etc. G. W. Wells, A.M., M.D., Medical Director Manhattan Life Insurance Co., Editor. Subscription, $2 a year. Club with TEXAS MEDICAL JOURNAL, both for $3.

---

**Home Industry.**—We see by the *Detroit Journal*, of Feb. 9, that a reporter of that paper has made a tour of Parke Davis & Co.'s laboratory, and has given their antitoxine industry a voluntary and complimentary write up. The *Journal* describes the mode of manufacture in a most realistic way, and states that the antitoxine will be sold early in the spring. Parke Davis & Co. are surely wide-awake, enterprising chemists, and are usually the first to put upon the market a new drug, or a new discovery in therapeutics—thus seconding the efforts of the investigators in medical science.

---

**Professor Isadore Dyer,** of Tulane University Medical Department, New Orleans, has resigned the editorship of the Department of Dermatology in this JOURNAL on account of press of professional duties. In this connection we must state that the same cause is operating to prevent the other members of the JOURNAL'S staff of collaborators from contributing their usual quota to the several departments, but we are advised that upon the close of the college term they will all resume the pen; and this summer we will give the readers of the Red Back a most acceptable variety of medical reading. Bear with us.

---

**To Prevent Blindness.**—The subject of the prevention of blindness by timely precaution has been agitated for a year or so, and it has culminated in steps to put the theory into practice. The Ohio legislature has passed a law requiring physicians and midwives to give timely attention to all infantile sore eyes, and Texas is following in the same line. A bill to prevent blindness is now before the legislature and probably will be passed. It requires midwives to report to a physician every case of sore eyes in the new born, and requires physicians to report to the health authorities those cases of a contagious nature.

P. S.—The committee reported adversely, and the bill will not pass.

---

**Dr. F. S. White,** so long and so favorably known to the profession of Texas as the able Superintendent of the State Lunatic Asylum at Austin, has located in Houston and will engage in general practice. The JOURNAL is pleased to learn that he has formed a co-partnership with Dr. E. T. Cook, a leading physician of the place. Dr. Cook is County Physician and President of the District Medical Society, and is well known throughout the State. Dr. White still has it in contemplation, as we announced in a recent issue—at some future time—to establish a sanitarium for the treatment of nervous diseases—a line in which he has had such long experience and such splendid results, but will postpone it until times get better. The Houston people are to be congratulated upon the accession of Dr. White and his estimable lady to their society and citizenship.

**Bills and Bills.**—There is now before the Texas legislature, in addition to other bills of interest to doctors, mentioned elsewhere, a bill to "regulate the practice" of pharmacy. It prohibits the refilling of a physician's prescription without written authority, where the prescription calls for opium, cocaine, morphine, etc.; a bill to "regulate the practice" of dentistry, requiring examination before a Dental Board; a bill to create a "State Embalming Board" to "regulate the practice" of embalming, in the interest of public health. There is also a bill to make appropriation for the payment of a large amount of quarantine bills, incurred under Gov. Ross' administration, over and above the appropriation for quarantine purposes. It amounts to about $70,000. The annual appropriation was then about what it is now, forty to fifty thousand dollars. Since which time the total expenses of quarantine have been kept within the appropriation.

---

**"Health Sanitation and Climatology of the Southern States"** is the name of the latest addition to our long list of medical (and allied) journals. We have received Vol. 1, No. 1. It is in a pretty, salmon colored cover, contains thirty pages, and is illustrated by pictures of scenery along the French Broad River. Dr. W. C. Murphy, Washington, D. C., is editor and publisher. [It has a pretty long name; we don't know what the doctor calls it when speaking of it, but suggest for convenience "the Journal of H. S. & C. of the S. S.]

Publications of this branch are getting to be very numerous. It shows increased interest in it which is very gratifying. Preventive medicine will be the medicine of the 20th century; most of the progress now being made is along the line of research as to causation. Means of prevention will naturally follow. The public is being slowly but surely educated up to an appreciation of the possibilities of State medicine. Dr. Murphy says in his salutatary: "The time will come when an educated public opinion will unite with the advances in medical science in demanding statutory provisions for disease, infection and the spread of epidemics as much as those which now govern our finance, commerce and property rights." And again: "Sanitarians estimate that if one half the money expended in *curing* consumption [Who "cures" it, doctor? Nobody pretends to cure it but Amick, we believe.—Ed.] were devoted to its *prevention*, the disease, within the next twenty years, could be effectually stamped out."

---

**Texas State Medical Association—Section on Surgery.**

Fort Worth, Texas, Feb. 25, 1895.

Dear Doctor—The time is rapidly approaching when the Texas State Medical Association will convene at Dallas.

It is earnestly desired that the Surgical Section be sustained at its former high standard.

We cordially invite you to contribute a paper upon some surgical subject, and advise us at your earliest convenience as to the title of same, that it may appear in the official program and receive its deserved consideration.

A. C. WALKER, M. D., Ch'n. Sec. Surgery.

E. D. CAPPS, Secretary, 514 Main street.

